# Dexmedetomidine reduces acute lung injury caused by LPS through the SIRT3 signaling pathway *in vivo*

**DOI:** 10.3389/fphar.2025.1524219

**Published:** 2025-06-25

**Authors:** Jian Chen, Yang Cai, Xiaochun Peng, Yuanling Xu, Liying Chen, Xinxin Pan, Yingying Sun

**Affiliations:** ^1^ Department of Anesthesiology, Anhui Provincial children’s Hospital, Anhui, China; ^2^ The Fifth Clinical Medical College of Anhui Medical University, Anhui, China; ^3^ The Children’s Medical Center of Anhui Medical University, Anhui, China

**Keywords:** acute lung injury, sirt3, dexmedetomidine, inflammation, oxidative stress

## Abstract

Acute lung injury (ALI) is a clinical syndrome characterized by excessive inflammatory responses. Despite the exploration of various therapeutic approaches, no effective pharmacological treatment is currently available for ALI. In the current study, we investigated the role of SIRT3 in LPS-induced ALI and the potential protective effects of dexmedetomidine (Dex), an agent that activates α2-adrenergic receptors. Histological analysis showed extensive lung damage and increased inflammatory cells in LPS-treated lung samples, with elevated TUNEL+ cells indicating apoptosis (*p* < 0.05). SIRT3 mRNA and protein expression were significantly downregulated following LPS treatment, both *in vivo* and *in vitro* (*p* < 0.05). DEX administration restored protein SIRT3 levels and reduced inflammation, while the SIRT3 inhibitor 3-TYP negated these benefits (*p* < 0.05). Additionally, DEX reduced pro-inflammatory cytokine levels and oxidative stress, effects that were also diminished by 3-TYP (*p* < 0.05). Our findings suggest that DEX exerts its protective effects against LPS-induced ALI via modulation of the SIRT3/LKB1/AMPK signaling pathway, highlighting the critical role of SIRT3 in inflammatory and oxidative stress responses in ALI.

## Introduction

Acute lung injury (ALI) represents a significant burden in critical care medicine, identified by its high incidence and the profound impact on patient morbidity and mortality (Liu C. et al., 2022; Liu X. et al., 2021). ALI is a clinical syndrome associated with excessive inflammatory responses, which can lead to diffuse alveolar damage, hypoxemic respiratory failure, and significant mortality rates (Yin et al., 2021). The condition is often induced by various factors, including sepsis, pneumonia, and severe trauma, and is marked by the disruption of the alveolar-capillary barrier, leading to protein-rich fluid exudation and impaired gas exchange (Matthay and Zemans, 2011). ALI progression to acute respiratory distress syndrome (ARDS) and the associated increase in mortality rates highlight the urgent need for effective treatments (Xu et al., 2024). The economic and healthcare system burden is substantial, with ALI/ARDS patients often requiring prolonged intensive care, mechanical ventilation, and other resource-intensive interventions (Rubenfeld et al., 2005). Despite the advancements in intensive care, the available therapies for ALI/ARDS remain largely supportive, such as mechanical ventilation, and do not adequately target the underlying pathophysiological mechanisms (Bellani et al., 2016). The development of gene therapy and the use of therapeutic nucleic acids, such as those discussed in the review by Zhuang et al. (2023), represent a promising direction in modulating the inflammatory response in ALI/ARDS. These approaches aim to target specific genes and molecular pathways involved in the disease process, offering a more precise and potentially more effective treatment modality. Thus, understanding the pathogenesis of ALI/ARDS is critically important for developing targeted therapies to effectively prevent or treat the condition.

SIRT3 has been implicated as a potential therapeutic target in various diseases, including neurodegenerative disorders and cardiovascular diseases (Cao et al., 2022). SIRT3 increases the activity of pyruvate dehydrogenase (PDH) through deacetylation, promoting glucose oxidation and thus playing a role in function and energy production (Chen et al., 2021). SIRT3 deficiency has been associated with proliferation, oxidative stress, inflammation, and fibrosis (Chelladurai et al., 2021). SIRT3/LKB1/AMPK signaling pathway is a key regulator of cellular metabolism and inflammation (Guo et al., 2022). Overexpression of SIRT3 has been shown to increase autophagy level and promote LKB1 phosphorylation, leading to the activation of AMPK and decreased phosphorylation of mTOR, suggesting a role for the LKB1-AMPK-mTOR pathway in the induction of autophagy (Zhang M. et al., 2018). SIRT3 has been reported to be associated with ALI, for example, SIRT3-p53 pathway in sepsis-associated ALI (Gao et al., 2024). However, the role of SIRT3/LKB1/AMPK signaling pathway in ALI remain to be supplemented.

Dexmedetomidine (DEX), an agonist of the α2-adrenergic receptor, is well-known for its properties that reduce inflammation, prevent apoptosis, and provide antioxidant effects. These characteristics have demonstrated considerable advantages in alleviating lung inflammation across different experimental models (Zhang H. et al., 2019; Li et al., 2018; Wang et al., 2019). DEX has the potential to mitigate sepsis-related ALI by influencing macrophage efferocytosis via the ROS/ADAM10/AXL signaling pathway (Li et al., 2024) and our previous results showed that DEX alleviates LPS-induced acute lung injury in rats (Sun et al., 2020). On the other hand, DEX has been shown to ameliorate cardiac ischemia/reperfusion injury that can be improved by promoting autophagy via the AMPK/SIRT3 signaling pathway activation (He et al., 2023), indicating the relationship between DEX and SIRT3 signals. However, whether DEX regulates SIRT3/LKB1/AMPK signaling pathway to modulate ALI needs further exploring.

Herein, the present study aimed to investigate the protective mechanisms of DEX in LPS-induced ALI. Our findings demonstrate that DEX exerts its anti-inflammatory effects through activation of the SIRT3/LKB1/AMPK signaling pathway, thereby providing novel mechanistic insights into its therapeutic potential for ALI and other inflammation-related disorders.

## Methods and materials

### Animals and ethics

The studies involving animal participants were reviewed and approved by the Ethics Committee of Anhui Medical University. Seven-day-old pathogen-free Sprague-Dawley (DS) rats (male, weighing 250–300 g) were sourced from the Experimental Animal Center of Anhui Medical University (license no. SCXK-2018–031). The rats were housed in standard laboratory cages under controlled conditions, maintaining a 12-h light/dark cycle at 22°C ± 2°C, with free access to food and water.

Sixteen rats were randomly divided into four groups (4 rats/group): a saline control group, a group exposed to LPS (#L2630-100 MG; Sigma Aldrich, United States), a group exposed to both LPS and Dex (#MB4091; Meilunbio Co., Ltd.; 25 μg/kg for rats according to previous reports (Sun et al., 2020; Wang et al., 2018)), and a group treated with LPS and Dex and 3-YTP, SIRT3 inhibitor (#HY-108331; Med chem express, Shanghai, China; 50 mg/kg, ip). The rats were anesthetized through an intraperitoneal injection of pentobarbital sodium at a dose of 40 mg/kg (Merck KGaA; cat. No. 1063180500). Tracheal intubation was performed using a micro-atomizer; the control group received 300 µL saline, whereas the LPS group was given 5 mg/kg LPS. Nebulized solution was given at an oxygen flow rate of 4 L per minute for 25 min. Animals were then fed normally for 30 min without intubation following every 6-hour period. Animals were euthanized by decapitation 12 h later. Lung tissues or bronchoalveolar lavage (BAL) were collected for evaluation, followed by a thoracotomy. The right lung tissue samples were isolated for further experiments.

### IHC analysis for lung morphology

For the morphological evaluation of the lungs, freshly collected samples were first weighed to record their wet mass. The samples were then dried overnight at 75°C to assess their dry mass (Herriges and Morrisey, 2014). Hematoxylin/eosin (HE) staining was performed to examine the histological structure of the lungs and assess the inflammatory response after LPS exposure. At designated time points, lung tissues were harvested and fixed in 4% paraformaldehyde, then dehydrated and embedded in paraffin wax. The samples were subsequently sectioned into 5 µm slices. The sections were analyzed using a fluorescence microscope (Olympus IX50; Olympus Corporation) in conjunction with analyzing software (NIS-Elements F3.2). Airspace volume density was determined by dividing the total airspace area by the total area (Plosa et al., 2014). At each time-point, at least 3 randomly selected images from 5 samples per group were analyzed.

### Biochemical analysis assay

The levels of SOD, CAT, and MDA were measured in both serum and lung tissue samples, using purchased kits (Beyotime, Shanghai, China). Serum concentrations of tumor necrosis factor-α (TNF-α, ab46070; Abcam), interleukin-1β (IL-1β, # BMS630; Thermal Fisher Scientific, Waltham, MA, United States) and IL-10 (#ERA23RB; Thermal Fisher Scientific), IL-8 (#EK720269; AFG Scientific, MA, United States) and IL-12 (#EK720274; AFG Scientific), IL-18 (#RAB1147; Merck, United States) and IL-6 (#RAB0311; Merck, United States), IL-5 (#ab267811; Abcam) and IL-17 (#ab119536; Abcam) were quantified using ELISA kits accordingly. A hydrogen peroxide assay kit (#S0051; Beyotime) was applied to measure the levels of hydrogen peroxide (H2O2) and the lucigenin chemiluminescence method was applied to determine the superoxide anion (O2-) levels in lung tissue samples. Briefly, lung tissue samples were homogenized and supernatant after centrifugation was incubated with 5 μM lucigenin in buffer, and then samples were recorded using a Tecan Infinite 200 by light emission. Addition incubation into the medium of 350 U/ml SOD were used to confirm the specificity for O2-. Protein concentrations were determined using a BCA Protein Concentration Assay Kit (Enhanced; #P0010; Beyotime).

### Inflammatory cell counts of bronchoalveolar lavage fluid (BALF)

Following treatment, animals were sacrificed, and BALF was obtained by washing the lungs three times with PBS via a tracheal cannula. The samples were centrifuged at 3,000 rpm for 10 min at 4°C, and the pellet of cells was resuspended in PBS for total cell count analysis using a hemocytometer. Cytospin preparations were made for differential cell count, which involved staining with Wright-Giemsa method. The proportions of macrophages, neutrophils, and lymphocytes in the BALF samples were determined by measuring leukocytes under a light microscope.

### ELISA assay

Serum samples were collected from administrated animals, and then were subjected to ELISA assay, for the antibodies: TNF-alpha, IL-6, IL-5, IL-1beta, IL-18 and IL-17.

### TUNEL analysis

This assay was conducted using a one-step detection kit (#C1086; Beyotime) following the protocol. Tissue samples were deparaffinized in xylene, rehydrated by a graded series of ethanol concentrations, and then heated for antigen retrieval. Hydrogen peroxide of 3% was used to inhibit endogenous peroxidase activity. Subsequently, different dilutions of terminal deoxynucleotidyl transferase in reaction buffer, containing a constant concentration of digoxigenin-labeled nucleotides, were applied to the sections and incubated at 37°C for 1 h, followed by a 10-minute incubation in Stop/Wash buffer. After extensive washing, 50 μL TUNEL detection dilution was applied for 30 min. Then the sections were mounted, and images were captured under the laser wavelength between 450 and 500 nm. The percentage of TUNEL+ cells was quantified and normalized relative to the total number of cells for each group.

### RT-qPCR assay

Total RNA was extracted from fresh samples using the Total RNA kit (Omega Bio-Tek, Inc.) and stored on ice, following the manufacturer’s protocol. First-strand cDNA synthesis and SYBR^®^ Green qPCR were conducted with the PrimeScript™ RT Reagent kit (Takara Bio, Inc.). Specific primers used in the study for *SIRT3* are listed as follows: F-5′-AAGACATACGGGTGGAGCCT -3′, R-5′ GGA​CTC​AGA​GCA​AAG​GAC​CC-3’. Real-time PCR was conducted using the SYBR Green kit (TINGEN Biotech, Beijing, China) under the following conditions: denaturation at 95°C for 20 s, annealing at 58°C for 20 s, and extension at 68°C for 30 s mRNA expression levels were measured by the 2^−ΔΔCq^ method and normalized to β-actin level. Levels of *SIRT3* in control group against β-actin were normalized to 100%. The qPCR results represent three independent experiments.

### Western blotting assay

This assay was conducted using a standard protocol with polyclonal antibodies specific to SIRT3, LKB1, phosphorylated(p)-LKB1, AMPK and phosphorylated(p)-AMPK. The procedures for protein extraction and immunoblotting were previously detailed. Protein samples were extracted from lung tissue homogenates using RIPA buffer (Beyotime, Shanghai, China), supplemented with inhibitors of protease and phosphatase. Protein concentrations were then determined using the BCA assay. The proteins were separated using 10% SDS-PAGE and subsequently transferred to a PVDF membrane. Following a blocking step with 5% non-fat milk, the membrane was incubated overnight at 4°C with antibodies targeting SIRT3 (1:1,000; #ab217319; Abcam), LKB1 (1:1,000; #ab199970), phosphorylated(p)-LKB1 (1:500; #ab63473; Abcam), AMPK (1:1,000; #ab32047; Abcam) and Anti-AMPK alpha 1 (phospho T183) + AMPK alpha 2 (phospho T172) antibody (1:1,000; #ab133448; Abcam) in TBS buffer. GAPDH was used as a loading control (1:2,000; #5174; CST, MA, United States). Following incubation with secondary antibodies, either HRP goat anti-rabbit (1:1,000; # A0208; Beyotime) or anti-mouse IgG (1:1,000; #A0216; Beyotime)—the blots were developed using the BeyoECL Star ECL (P0018AM; Beyotime) and imaged. Band intensities were quantified using ImageJ (NIH, United States). Levels in control group against GAPDH were normalized to 100%. The Western blot results represent three independent experiments.

### Statistical analysis

Statistical analyses and graph construction were conducted by the software package (version 10.0; GraphPad Software, Inc.). Data are expressed as mean ± standard deviation (SD) from a minimum of three independent experiments. To assess significant differences between control and treatment groups, one-way ANOVA followed by Tukey’s *post hoc* test or unpaired Student’s t-test were employed. A p-value of less than 0.05 was regarded as statistically significant.

## Results

### LPS induces acute lung injury (ALI)

**FIGURE 1:**
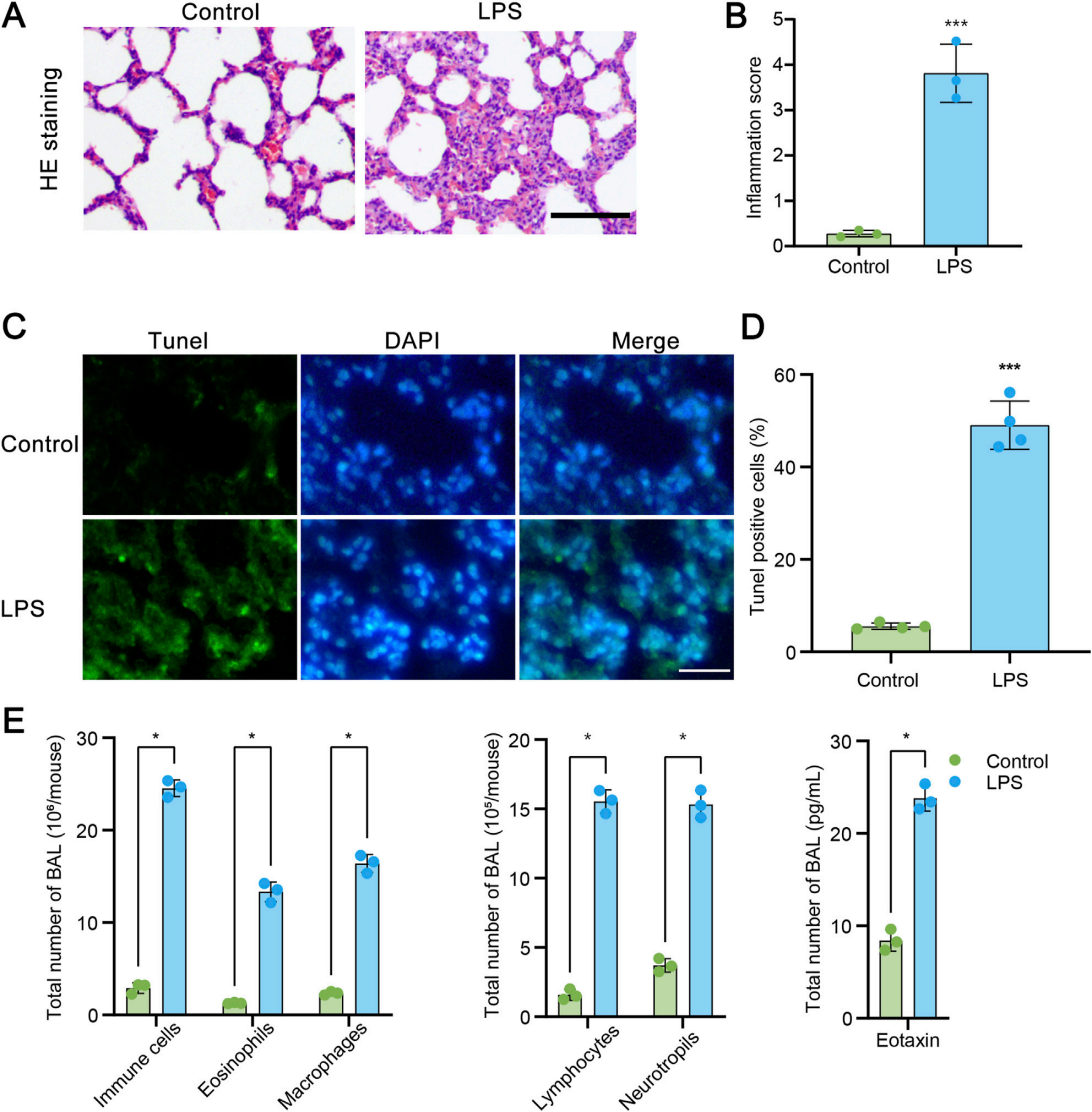
LPS administration induces to acute lung injury (ALI). **(A)** Hematoxylin and eosin (HE) staining of transverse lung sections from rats in the control and LPS-treated groups. **(B)** Quantification of the inflammatory response based on HE staining (n = 3). **(C)** TUNEL staining of transverse lung sections from the control and LPS-treated groups. **(D)** The number of TUNEL-positive cells relative to the total cells in lung tissue from the control and LPS-treated groups (n = 4). **(E)** Assessment of total cells, neutrophils, lymphocytes, macrophages, eosinophils, and Eotaxin levels in bronchoalveolar lavage fluid (BAL) by Wright-Giemsa (n = 3). Scale bars = 100 μm. Data are presented as mean ± SD. *P < 0.05 vs control; ***P < 0.001 vs control.

We first constructed a commonly used acute lung injury model using LPS. As described in the methods, rats were treated with LPS and then samples were collected and subjected to HE staining. Histological characteristics of the sections were observed. As illustrated in Figure 1A, LPS administration significantly caused the diffuse damage in the alveoli, alveolar tubes, alveolar sacs, bronchi, and alveolar septa. The quantified data of inflammation score shown in Figure 1B revealed that numerous inflammatory cells were observed in the alveolar septa after LPS exposure. Furthermore, samples were subjected to TUNEL assay. The results in Figure 1C showed that LPS administration markedly increased the number of TUNEL-positive pulmonary cells in lung samples. The quantified data were shown in Figure 1D. Then, we conducted bronchoalveolar lavages (BAL) assay to reveal the inflammatory cell infiltration by Wright-Giemsa method. As shown in Figure 1E, LPS administration significantly resulted in the increasement of immune cells, eosinophils, macrophages, lymphocytes and neutrophils. Furthermore, in LPS treated lung samples, the BAL eotaxin level was also increased. These data indicate that LPS administration significantly induced acute lung injury.

### Dexmedetomidine (DEX) regulates the SIRT3/LKB1/AMPK signaling pathway in LPS-induced acute lung injury

**FIGURE 2:**
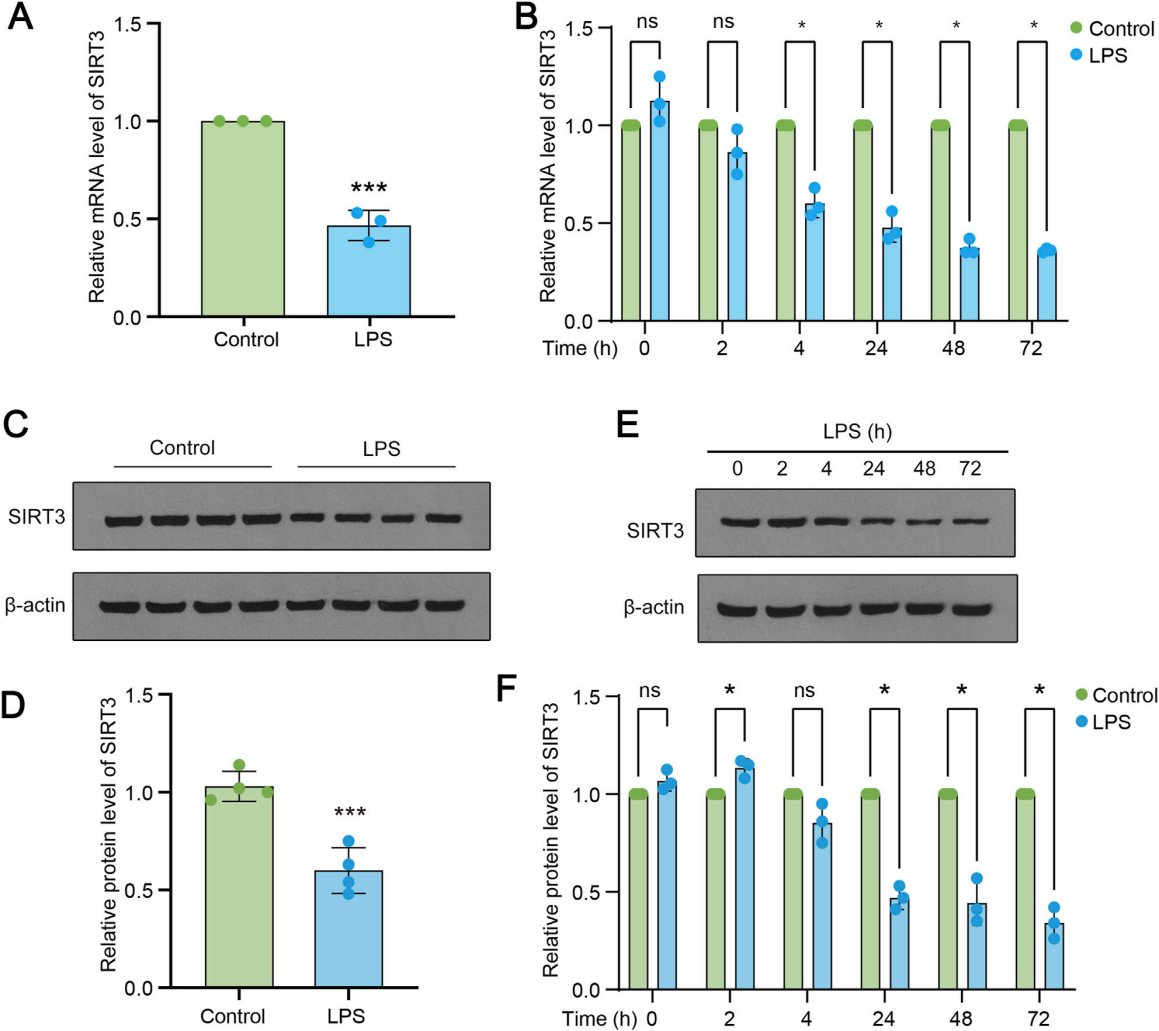
SIRT3 is downregulated during LPS induced ALI. **(A)** mRNA expression levels of SIRT3 in control and LPS-treated rats. **(B)** mRNA levels of SIRT3 in LPS-induced rat lung endothelial cells at 2, 4, 24, 48, and 72 h **(C–D)** Protein levels of SIRT3 in lung tissue samples (n = 4) from LPS-treated and control rats. **(E–F)** Protein levels of SIRT3 in lung endothelial cells at 2, 4, 24, 48, and 72 h (n = 3). Data are expressed as mean ± SD (n = 3 or 4). *P < 0.05 vs control; ***P < 0.001 vs control.

Next, we tried to ask whether SIRT3 was involved in LPS-induced ALI. Samples from LPS treated rats were subjected to RT-qPCR and the levels of SIRT3 were measured. As shown in Figure 2A, the levels of SIRT3 were significantly reduced in LPS group, compared to control rats. Then cultured lung epithelial cells were treated with LPS of indicated time points (2, 4, 24, 48 and 72 h). The data revealed that the mRNA levels of SIRT3 were gradually decreasing along the time points (Figure 2B). Samples of LPS treated rats and cultured cells administrated with LPS were subjected to Western blotting assay. The data showed that SIRT3 protein levels were also markedly decreased upon LPS treatment (Figures 2C,D). Also, the levels of SIRT3 protein were gradually downregulated in LPS-treated lung endothelial cells, same trend as the mRNA levels (Figures 2E,F).

**FIGURE 3:**
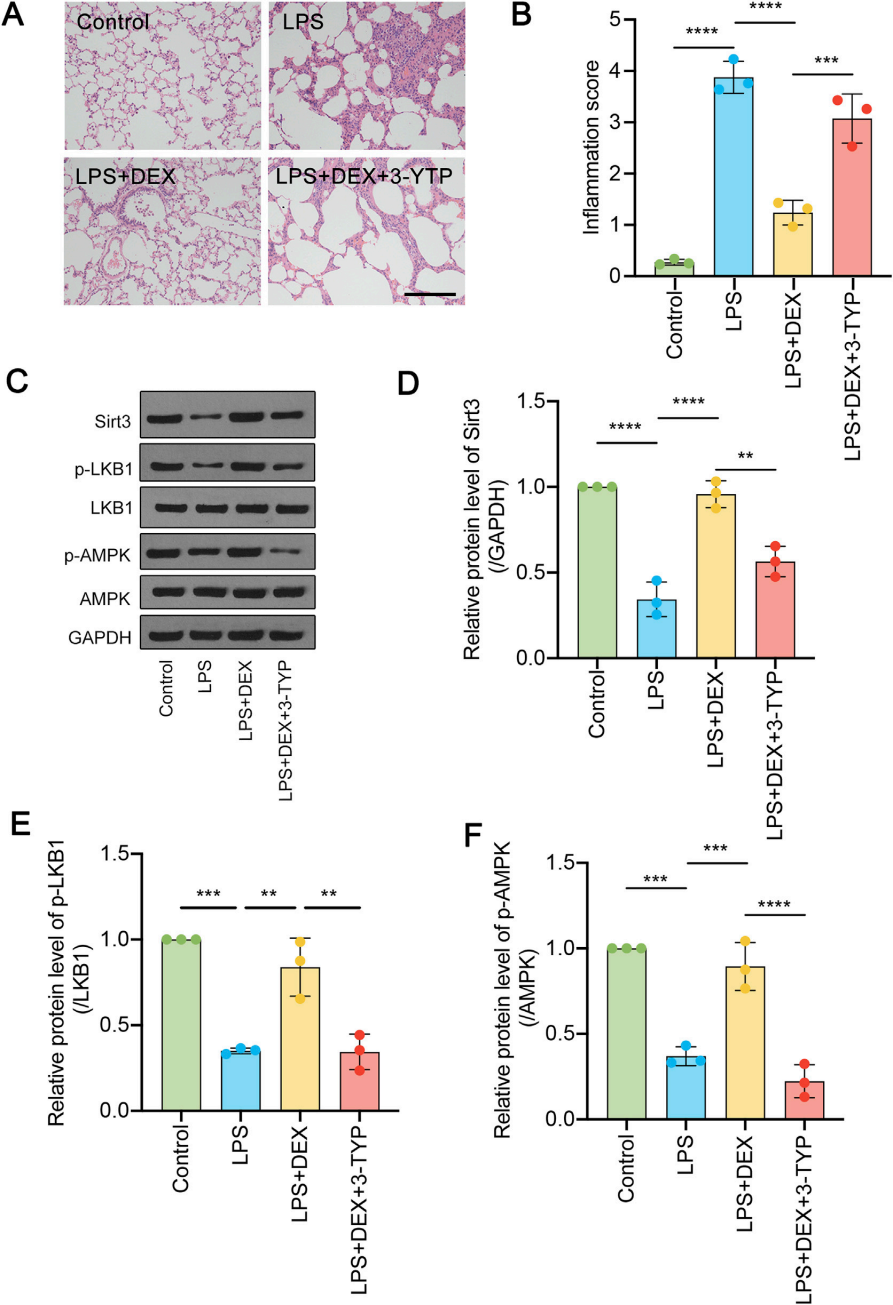
Dexmedetomidine (DEX) regulates the SIRT3/LKB1/AMPK signaling pathway in LPS-induced ALI. **(A)** HE-staining of transverse lung sections from rats in the control and LPS-treated groups, LPS + DEX, LPS + DEX+3-YTP (inhibitor of SIRT3) was performed. **(B)** The inflammation score was quantified for **(A)**. **(C)** The protein levels of total SIRT3, phosphorylated and total LKB1 and phosphorylated and total AMPK were determined. **(D–F)** The quantified data were shown. The data are represented as mean ± SD (n = 3). **P < 0.01; ***P < 0.001; ****P < 0.0001.

We have previously reported that DEX could alleviate LPS-induced acute lung injury (Sun et al., 2020). Since SIRT3 was involved in LPS-induced ALI, we asked whether DEX would function via the regulation of SIRT3 signaling pathway. LPS-administrated rats were treated with DEX, or the SIRT3 inhibitor 3-TYP. Firstly, we performed immunohistochemistry assay to reveal the histological characteristics of the sections under different treatment. As shown in Figure 3A, LPS-induced inflammation was significantly suppressed by DEX treatment, however, this process could be restored by SIRT3 inhibitor. The diffuse damage in the alveoli, alveolar tubes, alveolar sacs, bronchi, and alveolar septa showed the same trend as inflammation response, which was quantified in Figure 3B. Next, LPS administration significantly decreased the SIRT3 protein levels (Figures 3C,D), which could be rescued by the treatment of DEX. However, the addition of SIRT3 inhibitor 3-TYP further alleviated the restored level of SIRT3 protein (Figure 3B). Furthermore, when compared to the total level, the phosphorylated level of LKB1 and AMPK proteins were also shown the same trend as SIRT3 protein (Figures 3E,F). These results suggest that SIRT3 signaling pathway is involved in LPS-induced AKL, which could be modulated by DEX treatment.

### Inhibition of SIRT3 signaling alleviates the protective effect of DEX against pro-inflammatory cytokine release and oxidative stress during LPS-induced ALI

**FIGURE 4:**
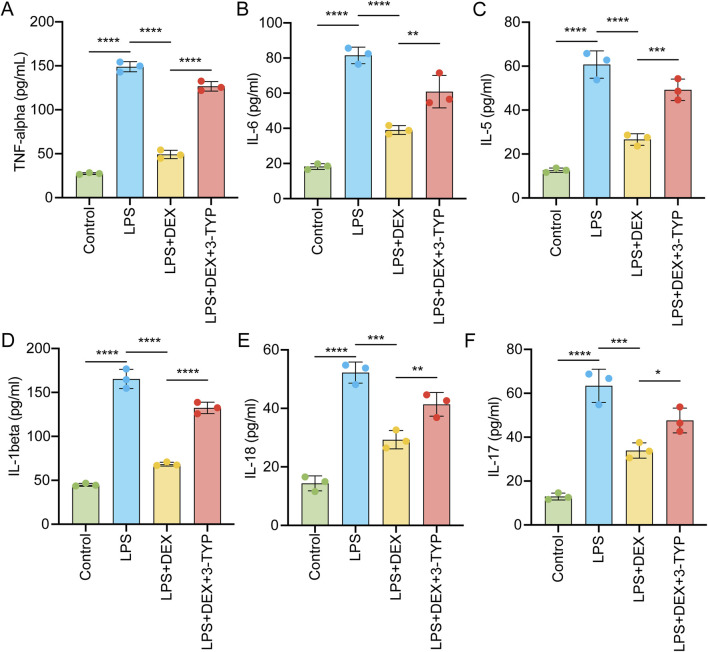
Inhibition of SIRT3 signaling abolishes the protective effect of DEX during pro-inflammatory cytokine release induced by LPS. Animals were treated as in Figure 3 and then serum pro-inflammatory cytokines were measured by ELISA, including **(A)** tumor necrosis factor-α (TNF-α), **(B)** interleukin-6 (IL-6), **(C)** IL-5, **(D)** IL-1β, **(E)** IL-18 and **(F)** IL-17. The data are presented as mean ± SD (n = 3). *P < 0.05; **P < 0.01; ***P < 0.001; ****P < 0.0001.

To investigate whether the pro-inflammatory response evoked by LPS could be mitigated by DEX through the SIRT3 signaling pathway, we administered rats with LPS, DEX, or the SIRT3 inhibitor 3-TYP. We then assessed the levels of pro-inflammatory cytokines in their serum. As depicted in Figure 4, exposure to LPS markedly upregulated the serum levels of TNF-α (Figure 4A), IL-6 (Figure 4B), IL-5 (Figure 4C), IL-1β (Figure 4D), IL-18 (Figure 4E), and IL-17 (Figure 4F). However, the administration of DEX notably attenuated these cytokine levels, an effect that was nullified by the additional treatment with 3-TYP (Figure 4). Collectively, these findings indicate that pharmacological inhibition of the SIRT3 pathway undermines the protective role of DEX in LPS-induced inflammation in ALI.

**FIGURE 5:**
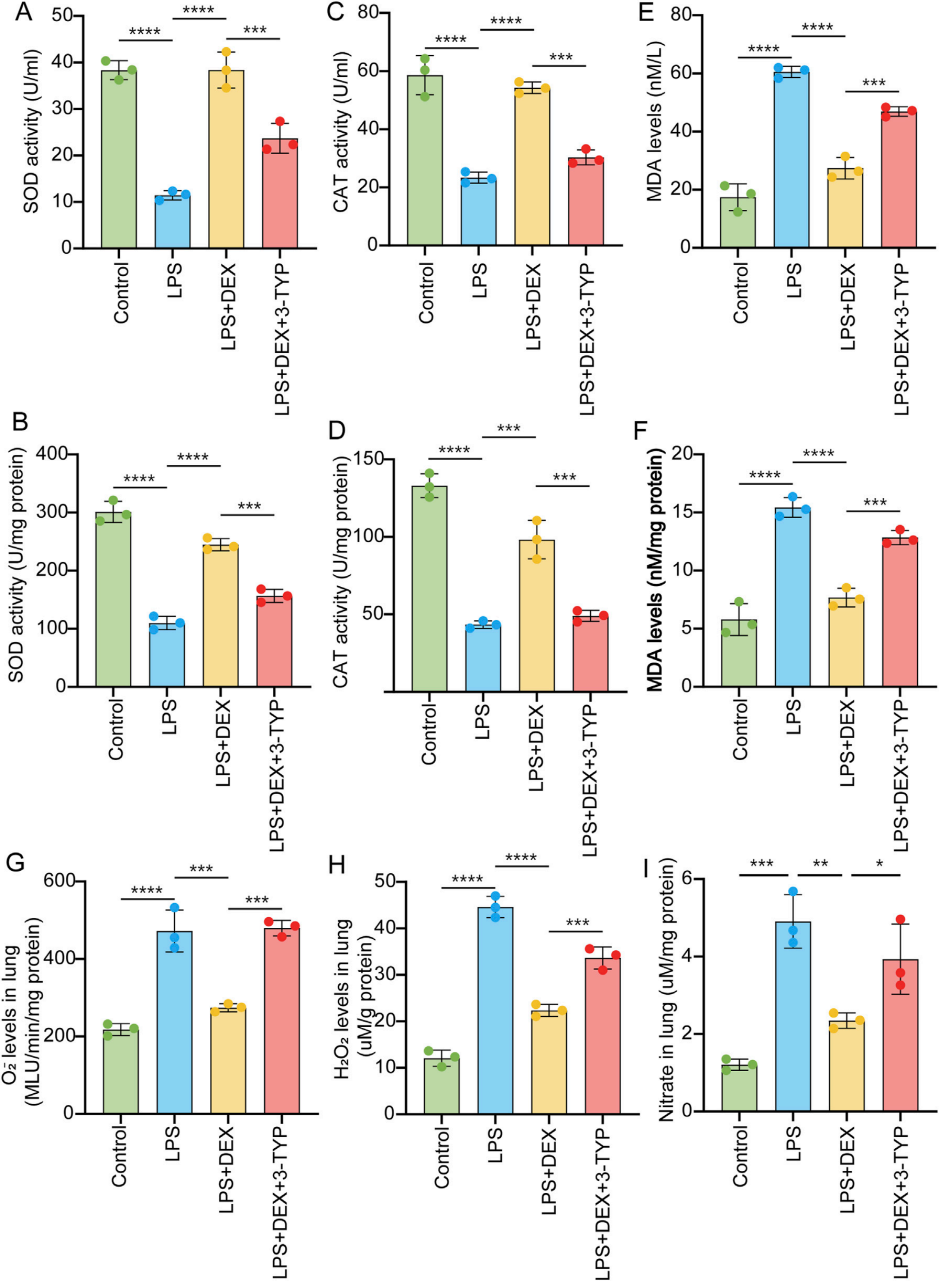
Inhibition of SIRT3 signaling abolishes the protective effect of DEX during oxidative stress induced by LPS. Rats were administered as in Figure 3. The collected samples were subjected to oxidative stress detection. SOD activity **(A,B)**, CAT activity **(C,D)** and MDA **(E,F)** levels measured in the serum or in the lung tissue samples of LPS-treated rats, with or without DEX or 3-YTP. O2- **(G)** and H2O2 **(H)**, and nitrate levels **(I)** in the LPS-induced lung tissue samples were measured. The data are presented as mean ± SD (n = 3). *P < 0.05; **P < 0.01; ***P < 0.001; ****P < 0.0001.

Oxidative stress, closely related to SIRT3 signaling pathway (Zheng et al., 2023), is critical for ALI (Zhong et al., 2024). To elucidate the role of SIRT3 in the generation of ROS in LPS-induced acute lung injury, we measured the activities of SOD and CAT in serum and lung tissue samples after LPS treatment. As depicted in Figures 5A–D, LPS induction significantly decreased the levels of SOD activity (Figures 5A,B) and CAT activity (Figures 5C,D). However, DEX administration notably reversed these reductions in SOD and CAT activities, an effect that was abrogated by the addition of the SIRT3 inhibitor 3-TYP (Figures 5A–D). In contrast, the level of MDA was upregulated by LPS treatment, but this increase was attenuated by DEX and further restored with the additional treatment of 3-TYP (Figures 5E,F). Moreover, we observed that the levels of oxidants such as O2−, H2O2, and nitrate in LPS-induced lung tissue samples were elevated following LPS treatment, but DEX treatment reduced these levels (Figures 5G–I). The addition of 3-TYP significantly inhibited the mitigating effect of DEX. Token together, these findings suggest that the inhibition of the SIRT3 pathway impairs the protective function of DEX against LPS-induced oxidative stress in ALI.

## Discussion

In general, we examined the effects of LPS on acute lung injury and the role of SIRT3 in mediating these effects. We established a rat model of ALI using LPS, observing significant damage to alveolar structures, increased inflammatory cell infiltration, and heightened apoptosis as indicated by TUNEL assays. Quantitative analysis revealed a marked upregulation of pro-inflammatory cytokines and a decrease in antioxidant enzyme activities, indicative of oxidative stress. We found that LPS administration resulted in a significant downregulation of SIRT3 at both the mRNA and protein levels in lung tissues and cultured epithelial cells. Treatment with DEX effectively restored SIRT3 levels and improved lung pathology. However, the use of the SIRT3 inhibitor 3-TYP undermined DEX’s protective effects, leading to elevated pro-inflammatory cytokines and oxidative stress markers.

ALI is characterized by an intense inflammatory response within the lung tissue. A systematic review and meta-analysis has indicated a significant association between ALI and elevated levels of certain inflammatory biomarkers, including angiopoietin-2 (ANG-2), interleukin (IL)-1β, IL-6, and tumor necrosis factor (TNF)-α (Liu Z. et al., 2022). These biomarkers are thought to play a crucial role in the pathogenesis of ALI, contributing to the severity of the condition and the subsequent development of acute respiratory distress syndrome (ARDS) (Mokra and Kosutova, 2015). The interaction between immune cells and vascular endothelial cells is another key aspect of the inflammatory process in ALI. These interactions lead to chemotaxis and adhesion of immune cells, which can exacerbate lung injury (Wu et al., 2022). For example, neutrophils, a type of white blood cell, migrating to the site of injury is a critical aspect of ALI pathophysiology, leading to further tissue damage and inflammation (Li et al., 2023). Furthermore, oxidative stress is intricately linked with acute lung injury, as it can lead to cellular damage, inflammation, and the exacerbation of lung tissue damage through the production of reactive oxygen species and the activation of inflammatory pathways (Bezerra et al., 2023). Oxidative stress not only causes direct tissue damage but also upregulates multiple inflammatory cytokines, perpetuating a cycle of damage and inflammation (Liu et al., 2023). Oxidants, such as ROS, act as inflammatory signaling molecules, activating pathways like NF-κB and NLRP3, which exacerbate ALI/ARDS (Ward, 2010). Here, we used LPS to construct the ALI model in rats. As we previously reported (Sun et al., 2020), LPS administration significantly induced severe damage in rat lungs (Figure 1). However, the detailed mechanism underlying inflammatory response during ALI need to be further explored.

The SIRT3/LKB1/AMPK signaling pathway plays a critical role in the regulation of cellular metabolism, energy homeostasis, and stress response, including its relationship with acute lung injury (ALI). SIRT3, a mitochondrial deacetylase, regulates proteins that are involved in ROS production, primarily by altering the acetylation of SOD2, which increases its activity and influences ROS homeostasis (Xi et al., 2024). SIRT3 abolishes sepsis-induced ALI via pyroptosis inhibition, which are crucial in mitigating inflammation and oxidative stress in ALI (Wu et al., 2023). Melatonin protects against ALI via SIRT3-dependent deacetylation of SOD2 (Ning et al., 2022). SIRT3 inhibition exacerbates mitochondrial dynamic imbalance and pro-inflammatory polarization, aggravating sepsis-induced ALI (Sun et al., 2024). Here, we found that LPS administration markedly downregulated the mRNA and protein levels of SIRT3, phosphorylated LKB1, and phosphorylated AMPK in lung samples. Dex treatment rescued these protein levels but could be suppressed by the additional administration of SIRT3 inhibitor 3-YTP (Figure 3). This finding suggests that the SIRT3/LKB1/AMPK signaling pathway is protective in ALI, which is consistent with above reports.

Dexmedetomidine (DEX) has been studied for its potential protective effects on ALI. Dexmedetomidine reduces systemic inflammation and lung injury by promoting Treg cells proliferation through the AMPK/SIRT1 signaling pathway (Zhang et al., 2023). DEX reduces LPS-induced ALI by targeting miR-381/NLRP3 axis (Zhang et al., 2018b). DEX mitigates IL-17-induced lung injury through a dose-dependent anti-inflammatory effect (Zhang et al., 2018c). DEX mitigates hyperoxia-induced ALI by inhibiting the activation of the NLRP3 inflammasome (Zhang et al., 2017). DEX protects against lung injury by influencing mitochondrial dynamics and promoting oxygen consumption (Zhang JR. et al., 2019), or via the PKC-alpha/HO-1 pathway (Song et al., 2022). DEX alleviates LPS-induced ALI in rats by regulating Nrf2/Keap1 and Akt signals (Yan et al., 2017). DEX alleviates pulmonary edema in LPS-induced ALI by upregulating AQP1 and AQP5 expression (Jiang et al., 2015). On the other hand, SIRT3 is involved in DEX regulated diseases. For example, DEX helps protect enteric glial cells from mitochondrial damage and cell death during experimental intestinal ischemia/reperfusion injury by utilizing a SIRT3-dependent pathway (Zhang et al., 2021). DEX safeguards the heart against ischemia-reperfusion injury by boosting autophagy via the AMPK/SIRT3 pathway, thereby reducing oxidative stress, inflammation, and improving cardiac function and mitochondrial integrity (He et al., 2023). DEX protects against nephritis by upregulating SIRT3 expression, which mitigates inflammation, oxidative stress, and apoptosis in renal cells both *in vivo* and *in vitro* (Lu et al., 2024). Here, in the current study, we found that DEX could also alleviate the LPS-induced inflammation and also DEX modulate SIRT3 signaling pathway to execute the protective function during ALI.

Dexmedetomidine is associated with several side effects, particularly hemodynamic and respiratory complications, which clinicians must monitor closely. Dexmedetomidine is associated with a significant decrease in heart rate compared to propofol, indicating potential bradycardia as a side effect (Nicholson et al., 2021). Dexmedetomidine has been noted to cause respiratory depression and hypoxia, particularly in combination with other sedatives (Liu S. et al., 2021). Despite its favorable respiratory profile, dexmedetomidine may still lead to severe circulatory complications in adults (Su and Hammer, 2011). The combination of dexmedetomidine with propofol in younger patients was associated with reduced mortality, while increasing dexmedetomidine doses correlated with increased mortality (Shehabi et al., 2023). While it shows efficacy in sedation and analgesia, its safety profile compared to other sedatives like propofol and olanzapine suggests a need for careful consideration in its use, especially in vulnerable populations.

In conclusion, the present study suggests that DEX mitigates LPS-induced ALI primarily through the SIRT3/LKB1/AMPK signaling pathway, reinforcing the importance of SIRT3 in the inflammatory and oxidative responses associated with ALI. These findings may offer insights into therapeutic strategies targeting SIRT3 in lung inflammatory diseases.

## Data Availability

The original contributions presented in the study are included in the article/supplementary material, further inquiries can be directed to the corresponding author.
